# Glucose Metabolism Evaluated by Glycated Hemoglobin and Insulin Sensitivity Indices in Children Treated with Recombinant Human Growth Hormone

**DOI:** 10.4274/jcrpe.galenos.2019.2019.0281

**Published:** 2019-11-22

**Authors:** Maria Chiara Pellegrin, Daria Michelon, Elena Faleschini, Claudio Germani, Egidio Barbi, Gianluca Tornese

**Affiliations:** 1 Institute for Maternal and Child Health, IRCCS Burlo Garofolo, Trieste, Italy; 2 University of Trieste, Trieste, Italy

**Keywords:** Glucose metabolism, growth hormone treatment, children, insulin sensitivity, glycated hemoglobin

## Abstract

**Objective:**

To evaluate glucose metabolism and insulin sensitivity in children with idiopathic growth hormone (GH) deficiency, treated with recombinant human GH (rhGH), and to identify possible risk factors for the development of glucose abnormalities in this population.

**Methods:**

We retrospectively collected data from 101 patients (60 males, median age 10.4 years, 77 prepubertal), with confirmed GH deficiency, enrolled before starting rhGH and followed up during the first three years of treatment. Glucose metabolism was evaluated annually by oral glucose tolerance test (OGTT) and glycated hemoglobin A1c (HbA1c). OGTT was used to calculate insulin sensitivity (HOMA-S) and insulin resistance (HOMA-IR), defined as HOMA-IR >3.

**Results:**

RhGH was effective in improving growth and dosages significantly reduced after the first year of therapy. No patient developed diabetes mellitus. After one year of therapy, a significant increase in HbA1c (p=0.0042) and insulin levels (fasting p<0.0001, 60 min p=0.0018, 120 min p=0.0003) was observed, with a higher prevalence of IR (p<0.05). These indices did not alter further during the follow-up and were not related to GH dose or to family history of diabetes. A significant correlation was found only for IR indices and pubertal status, weight and age (p<0.05).

**Conclusion:**

In this retrospective study on a large GH deficient pediatric population, conventional use of replacement therapy resulted in an increase in HbA1c and IR after one year of therapy, regardless of rhGH dosage. These alterations did not worsen significantly in the following two years and were not associated with overt diabetes.

**What is already known on this topic?**

Growth hormone (GH) has an anti-insulin effect. Children treated with recombinant human GH (rhGH) may develop abnormalities in glucose metabolism and present a higher incidence of type-2 diabetes mellitus. This applies particularly to subjects with predisposing conditions such as obesity or positive family history.

**What this study adds?**

In this study, conventional use of rhGH, in a large GH deficient pediatric population, resulted in increased hemoglobin A1c and worsened insulin sensitivity after one year of therapy. However, at the subsequent follow-up, these indices had not deteriorated further and were not associated with significant changes in glucose metabolism. This therapy proved to be safe, even in subjects considered at risk for glucose metabolism alteration.

## Introduction

Growth hormone (GH) exerts a variety of different metabolic actions, including playing a role in glucose homeostasis (1). It contributes to maintaining normoglycemia and is considered an insulin antagonist, especially during fasting, via stimulation of hepatic gluconeogenesis and suppression of insulin-mediated glucose uptake in peripheral tissues (2).

The benefits of human recombinant GH (rhGH) replacement therapy in improving height in children with GH deficiency (GHD) are well recognized (3). RhGH therapy can also improve body composition, lipid profile and bone mineral density (4). As regards carbohydrate metabolism, observational studies have reported an increased incidence of type 2 diabetes in GH-treated children (5,6). Although the incidence of type 2 diabetes is low (one case for every 3000 person-years of treatment), monitoring glucose levels before and, periodically, during treatment, has been recommended, especially in subjects with pre-existing risk factors such as obesity, positive family history of type 2 diabetes and pretreatment insulin resistance (IR) (5,6).

In terms of rhGH effects on insulin sensitivity, GH treatment leads to a compensatory increase in insulin secretion before the appearance of overt glucose abnormalities (1). Thus, decreased insulin sensitivity may be detected even without changes in glucose tolerance (7).

In recent years, a variety of different parameters and indices have been used to study the influence of GH treatment on glucose and insulin homeostasis (8). Biomarkers such as glycated hemoglobin A1c (HbA1c) and indices of glucose tolerance are now widely employed in the diagnosis and monitoring of patients with glucose abnormalities, but only one report explored their potential application in the field of rhGH therapy over a one year follow-up period (9).

The aim of our study was to evaluate the influence of GH replacement therapy on glucose metabolism and insulin sensitivity in a cohort of idiopathic GHD children over a three year follow-up period. The secondary aim was to identify risk factors that could predict the development of impaired glucose metabolism in this population.

## Methods

### Study Design

We retrospectively collected information on all the children consecutively diagnosed with isolated GHD at the Institute for Maternal and Child Health-IRCCS “Burlo Garofolo” (Trieste, Italy) between March 1^st^, 2007 and December 31^st^, 2013. The diagnosis of GHD was established based on the clinical, auxological and biochemical criteria set by AIFA (Agenzia Italiana del Farmaco, Italian Medicines Agency) at the time of first evaluation (10). Auxological and laboratory evaluations were collected before starting rhGH (baseline), and after one, two and three years of treatment. All patients were regularly followed-up every six months.

Height and body mass index (BMI) were expressed as standard deviation scores (SDS) based on the Italian reference growth charts (11) using Growth Calculator 3 Software (Weboriented.it. Growth Calculator 3). Pubertal status was assessed with Tanner staging.

Parents provided informed consent to obtain and store blood samples for research purposes, in accordance with the Declaration of Helsinki of 1975. The study was approved by the Institutional Review Committee of IRCCS “Burlo Garofolo” (approval number: RC 32/18 Line 2).

### Growth Hormone Treatment

Biosynthetic rhGH (Genotropin^®^, Humatrope^®^, Norditropin^®^, NutropinAq^®^, Omnitrope^®^, Saizen^®^, or Zomacton^®^) was administered once daily at bedtime, for a total of six or seven injections per week. Initial subcutaneous dose was 25-35 mcg/kg/day, which was gradually modified during the follow-up based on growth velocity and insulin-like growth factor-1 (IGF-1) concentration.

### Monitoring of Glucose Metabolism

Before starting GH treatment and every year at follow-up, monitoring of glucose metabolism was carried out on each patient, after an overnight fast: fasting glucose, fasting insulin and HbA1c were determined and an oral glucose tolerance test (OGTT) was performed (glucose load of 1.75 g/kg of body weight up to a maximum of 75 g). Blood samples for glycaemia and insulinemia were collected after 60 and 120 minutes (Glu60, Glu120 and Ins60, Ins120, respectively).

Altered glucose metabolism was defined according to the American Diabetes Association criteria for prediabetes (12) and included impaired fasting glucose (IFG), impaired glucose tolerance (IGT) or impaired HbA1c (39-47 mmol/mol, using IFCC method). Diabetes was diagnosed if fasting glucose was ≥126 mg/dL, or Glu120 was ≥200 mg/dL, or HbA1c was ≥48 mmol/mol. In the absence of unequivocal hyperglycemia, results were confirmed by repeat testing (12).

Hyperinsulinemia was diagnosed if fasting insulin was ≥15 µU/mL in prepubertal and ≥20 µU/mL in pubertal children or with Ins60 ≥150 µU/mL or Ins120 ≥75 µU/mL (13). We assessed basal insulin secretion by Homeostasis Model Assessment for β-cell function index (HOMA-β) and insulin sensitivity (HOMA-S) using the HOMA calculator (www.dtu.ox.ac.uk/homacalculator/. HOMA Calculator). IR was evaluated by Homeostasis Model Assessment Insulin Resistance index (HOMA-IR), applying the Matthews formula [fasting insulin (µU/mL) x fasting glucose (mg/dL)/405] (14). A diagnosis of IR was made if the HOMA-IR value was >3, in accordance with literature (15,16).

### Hormone and Biochemical Assays

All biochemical data were measured in our laboratory using standard methods. Glycemia was measured via a hexokinase enzymatic reaction by Cobas 501/502 (Roche Diagnostics, Indianapolis, IN, USA). Insulinemia was measured using an electrochemiluminescence immunoassay by Elecsys immunoanalizer and Cobas e (Roche Diagnostics, Indianapolis, IN, USA). HbA1c was assessed using turbidimetric inhibition immunoassay by Cobas Integra 400 Tina-quant Hemoglobin A1c Gen.2 (Roche Diagnostics, Indianapolis, IN, USA). Serum GH was assessed with a two-site chemiluminescent immunometric assay on the IMMULITE 2000 analyzer (Siemens Healthcare Diagnostics, United Kingdom, UK) with a sensitivity of 0.01 ng/mL. Serum total IGF-I was assayed using a solid-phase, enzyme-labeled chemiluminescent immunometric assay by IMMULITE 2000 (Siemens Healthcare Diagnostics, United Kingdom, UK) with a sensitivity of 20 ng/mL.

### Statistical Analysis

All statistical analyses were conducted with Stata/IC 14.2 (StataCorp LLC, College Station, USA). Data were described as frequencies and percentages or as medians and interquartile ranges, as appropriate. The Wilcoxon sign-rank test for paired samples was employed to compare repeated measures taken at two different points in time. Spearman correlations were used to compare the ranks of two continuous variables. The Mann-Whitney rank-sum test was carried out to compare unrepeated measures between two groups. The McNemar test was used to compare proportions for paired nominal data. Bivariate and multivariate logistic regressions were carried out to study associations between dichotomous outcomes and one or more independent variables. A p value <0.05 was considered statistically significant.

## Results

### Patient Characteristics

We studied 101 GHD-children (60 males). All children failed two GH stimulation tests, with GH peaks being below 10 ng/mL [first peak median (range) 6.20 (4.51-7.74); second peak median 6.41 (3.89-7.90)]. At baseline, 77 children (76.3%) were prepubertal (Tanner stage 1). Median (range) age at start of GH treatment was 10.4 (7.7-12.5) years.

The clinical and biochemical features of the population at baseline and after one, two and three years of therapy are shown in Table 1.

### Growth

At baseline, GHD children displayed short stature and low IGF-1 concentrations, as expected. A significant and consistent increase in height SDS and IGF-1, together with an increase in body weight SDS, was noticed over the study period (p<0.0001, see Table 1). BMI SDS did not change significantly until the second year of treatment, and subsequently increased during the third year (p=0.0133).

The dose of rhGH significantly decreased after the first year of treatment (p<0.0001) and remained stable in subsequent years. No correlations were found between the dose of rhGH during treatment and any of the other variables examined: age; height; puberty; and BMI.

### Evaluation of Glucose Metabolism

No patient developed diabetes mellitus during the study period.

**Figure 1:**
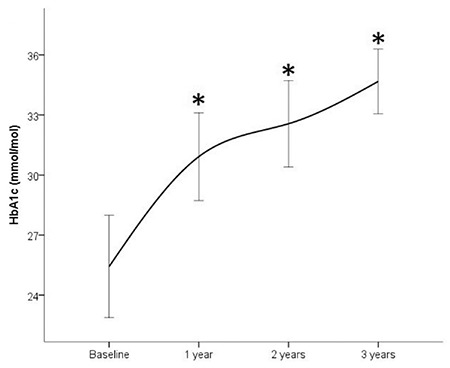
Glycated hemoglobin A1c during the three years of follow-up. *p<0.05 vs baseline HbA1c: hemoglobin A1c

HbA1c significantly increased after one year of treatment, from 25.5±11.9 mmol/mol to 30.9±9.9 mmol/mol (p=0.0042) and thereafter remained stable over the following years (second year 32.6±10.6 mmol/mol, third year 34.7±6.5 mmol/mol, Figure 1). Compared to baseline values, glycated hemoglobin was significantly increased during all three years of follow-up. The increase occurred mostly in the first year, continuing in the subsequent years but not so rapidly and without statistical significance.

**Figure 2:**
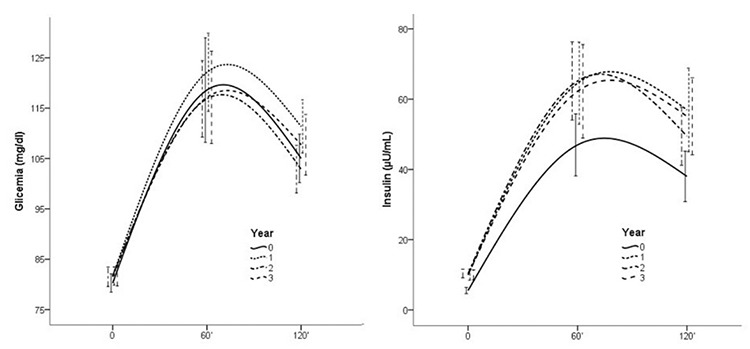
Time response of glucose and insulin levels during oral glucose tolerance test, at baseline and in the three years of follow-up. A significant increase was found only in insulin levels after the first year (see text for p values)

OGTT did not detect significant increases in glucose concentrations over the years, while a significant increase in insulin levels was found after the first year of treatment versus baseline, in fasting insulin (p<0.0001), and in the 60 (p=0.0018) and 120 minutes (p=0.0003) samples. Insulin concentrations were significantly correlated with age, BMI, IGF-1 and pubertal status, at baseline and in the follow-up period (Table 2). Along with an increase in insulin secretion, a significant increase in HOMA-B was observed (Table 1). No further significant increases were observed in the following years (Figure 2).

**Figure 3:**
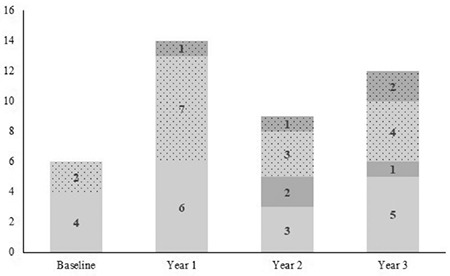
Number of growth hormone deficient children with glucose metabolism alterations before starting growth hormone therapy and in the follow-up. Dotted area: impaired hemoglobin A1c. Non-dotted area: impaired fasting glucose or impaired glucose tolerance. Light grey area: newly diagnosed glucose metabolism alterations. Dark grey area: alterations confirmed from the previous year

Before the therapy was started, alterations in glucose metabolism were detected in six (5.9%) patients, four presenting IGT and two presenting impaired HbA1c. In these patients, glucose metabolism normalized during the follow-up (only for one patient impaired HbA1c was confirmed after one year, but normalized in subsequent follow-ups). During the study period, IFG was present in five (5.0%) patients and 12 (11.9%) developed IGT. Glucose and HbA1c alterations were confirmed only occasionally in these patients during the follow-up period (Figure 3). These cases were managed with dietary and lifestyle advice, without stopping the treatment. In the 29 subjects with a positive family history of type 2 diabetes, the risk of developing glucose metabolism alterations was not increased when compared with the rest of the cohort.

In a multivariate logistic regression model that considered age, gender, BMI and pubertal status, none of the model variables was significantly associated with IFG, IGT or HbA1c.

### Insulin Resistance

A significant increase in HOMA-IR and decrease in HOMA-S were observed between baseline and the first year of treatment (Table 1). Prevalence of IR (altered HOMA-IR) increased from baseline to first year (from 0% to 6.9%, p=0.045), with a non-significant decrease in the second (1.2%) and third (4.6%) years.

Univariate analysis revealed that IGF-1 concentrations were significantly (p<0.01) and positively correlated with HOMA-IR and inversely correlated with HOMA-S. Weight and age were significantly (p<0.01) correlated with these indices (positively with HOMA-IR and inversely with HOMA-S). No correlation was found with BMI. As expected, HOMA-IR was significantly lower and HOMA-S significantly higher than baseline in pubertal children, after the first year.

## Discussion

Data from the main registries on children treated with rhGH (5,6) suggest that this therapy may accelerate the onset of type 2 diabetes mellitus in predisposed patients, with a prevalence of 0.36% of abnormal glucose metabolism and a six-fold increase in the incidence of type 2 diabetes. Several other studies have investigated the effects of rhGH therapy on glucose metabolism in children (17,18,19,20,21,22). Nevertheless, as highlighted in a recent systematic review (8), only in relatively few studies glucose metabolism abnormalities were the main outcome. The use of different methods to study glucose metabolism and the heterogeneity of the populations evaluated precluded the possibility of obtaining strong evidence of possible glycemic alterations caused by rhGH. The two largest case-control studies reported no significant increase in metabolic disorders, but presented conflicting results with low global clinical significance on the effects of rhGH on insulin sensitivity markers (19,22).

In this study, which included a large cohort of GHD children treated with conventional doses of rhGH for three years, therapy was well tolerated, without major changes in glucose metabolism occurring. No children developed overt diabetes mellitus. In line with previous data (9,19,23), we found an increase in HbA1c and insulin levels, HOMA-IR and HOMA-B values, with a concomitant decrease in HOMA-S. The significant increase in HbA1c, insulin concentrations and IR indices after the first year of treatment, compared to baseline, persisted in subsequent years of follow-up but did not significantly increase from one year to the next. Albeit non statistically significant and of little or none clinical impact, values were not ‘stable’ at three years, showing a slight but persistent increase. The lack of data after three years do not allow to define if this alteration is persistent or self limiting in a longer period. Larger studies with longer follow may help to better understand this issue.

The observed increase did not translate into significant alterations in either basal or OGTT-measured glucose metabolism: glucose abnormalities were only mild and transitory, and unrelated to rhGH doses, BMI or positive family history. This does not necessarily mean that GH administration does not increase glucose production by stimulating insulin secretion. As reported by Baronio et al (21), the enhanced insulin secretion observed in children with GHD might be not due to IR, but rather to a positive influence of GH treatment on β-cell secretory capacity. In our study, rhGH doses were maintained in the recommended range for isolated GHD (25-35 mcg/kg/day) (24) for the entire follow-up period. Even if the dosage was higher during the first year, when a significant impairment in HbA1c and in insulin sensitivity indices was observed, no significant correlation was found. Our data cannot answer the question of whether, for higher doses of GH, the effect of therapy in inducing IR and diabetes might be more significant.

Remarkably, 6% of patients presented with pretreatment alterations in glucose metabolism, but none of these patients developed diabetes, nor confirmed persistent alterations during treatment. This is in line with the results of Radetti et al (19), who observed a normalization of glucose tolerance in children presenting with IGT before starting rhGH treatment. We speculate that the increased linear growth and the likely improvements in lean body mass composition induced by GH replacement, may have ameliorated glucose metabolism in these patients.

Few studies have tried to identify predictive factors relating to glucose metabolism alterations in children treated with rhGH. The two largest studies (5,6) postulated that the most relevant predictors for the development of IR and diabetes are individual predisposition and presence of pre-existing metabolic risk factors such as obesity, family history of diabetes, pretreatment IR, previous cranial irradiation and glucocorticoid treatment. In the present study, the development of abnormal glucose metabolism, defined as IFG, IGT or impaired HbA1c, was not predicted by any of these factors. No correlation was found between rhGH dosage, positive family history for diabetes, BMI and presence of IFG, IGT or impaired HbA1c, although this conclusion is limited by the small number of detected cases.

### Study Limitations

The major limitation of this study is its retrospective nature. In addition, the gold standard method for the detection of insulin sensitivity, i.e. the euglycemic hyperinsulinemic clamp, was not used. Markers for the assessment of insulin secretion included fasting insulin and HOMA-B, while HOMA-IR was used as a surrogate estimate of insulin sensitivity. The fact that glucose metabolism was not re-evaluated after rhGH discontinuation is a further limitation of the study. Furthermore, a longer follow-up period would have been more informative in patients who needed to maintain therapy after the first three years. Therefore, the risk factors for the persistence of glucose abnormalities cannot be adequately analysed.

## Conclusion

In conclusion rhGH replacement therapy at recommended dosages may be considered safe in terms of metabolic effects. A significant increase in HbA1c and IR after one year of therapy was observed. These alterations persisted, but did not worsen significantly in the following two years and did not bear to overt diabetes in any case. Glucose abnormalities were infrequent and, in the majority of cases, not confirmed in the subsequent follow-up, even in the presence of pretreatment metabolic impairment. Therefore, pre-existing glucose metabolism alterations should not represent a limitation to starting rhGH therapy and new onset alterations during treatment should be appropriately managed by intervening on predisposing factors, rather than by modifying rhGH dosage.

## Figures and Tables

**Table 1:**
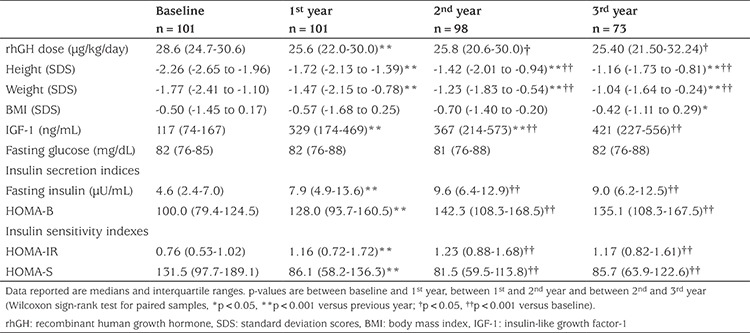
Clinical and biochemical features at baseline and at completion of first, second and third year of growth hormone treatment

**Table 2:**
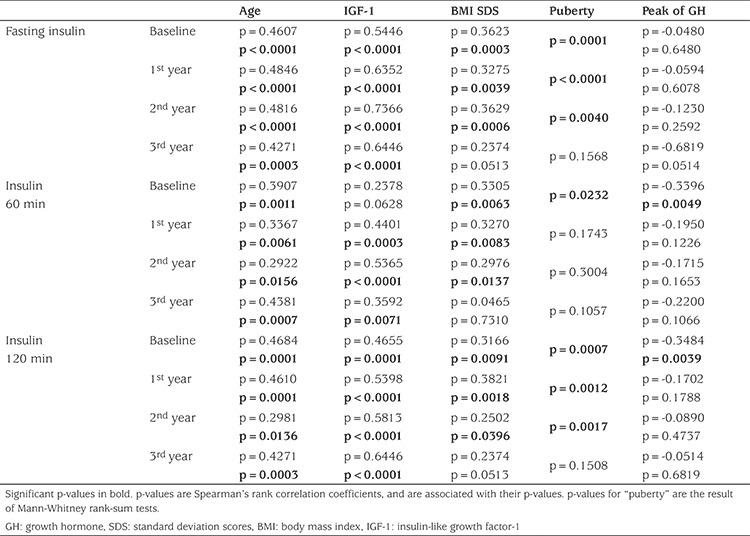
Bivariate analysis of the relation between insulinemia (fasting, at 60 minutes and at 120 minutes during oral glucose tolerance test) and relevant variables over the study period
